# Health service providers’ perspectives on the influence of modern health systems on adolescents’ sexual health practices in Umguza and Mberengwa districts of Zimbabwe

**DOI:** 10.1186/s12978-021-01314-5

**Published:** 2022-01-03

**Authors:** Wilfred Njabulo Nunu, Lufuno Makhado, Jabu Tsakani Mabunda, Rachel Tsakani Lebese

**Affiliations:** 1 Department of Public Health, School of Health Sciences, University of Venda, Thohoyandou, South Africa; 2 School of Health Sciences, University of Venda, Thohoyandou, South Africa; 3 Department of Environmental Science and Health, Faculty of Applied Sciences, National University of Science and Technology, Ascot, Corner Gwanda Road and Cecil Avenue, P O Box Ac 939, Bulawayo, Zimbabwe

**Keywords:** Adolescents, Health service providers, Modern health systems, Sexual health, Zimbabwe

## Abstract

**Background:**

Health service providers play a significant role in crafting and implementing health policies and programs that manage adolescent sexual health-related issues at different health system levels. These influence adolescent sexual behaviours and practices.

**Aim:**

This study explored the roles of health service providers in managing adolescent sexual issues and how this impacts their sexual behaviours and practices. The study further probed the health service providers on how the indigenous health system could be integrated into the modern health system for effective management of adolescent sexual health related issues.

**Methods:**

A qualitative cross-sectional survey was conducted on purposively selected health service providers in health facilities in Mberengwa and Umguza districts. Data was collected using unstructured interviews that were recorded, transcribed verbatim, and thematically analysed. Findings were presented as clearly defined as superordinate and subordinate themes.

**Results:**

A total of five superordinate themes and 19 subordinate themes emerged from the interrogated data. The superordinate themes were: overview of adolescent sexual health issues, role of modern health system in adolescent sexual health issues, challenges encountered, indigenous health system factors that could be factored into modern health systems, and strategies to foster the integration of indigenous health system and modern health system. The subordinate themes explored in-depth the findings of the key stakeholders under the five superordinate themes.

**Conclusions:**

From the findings, it can be concluded that health service providers play an essential role in shaping and providing adolescent sexual health services that adolescents utilise despite challenges that have reduced demand for these services. Therefore, there is a need to point out that there is a window of opportunity to foster collaborations between the indigenous health system and the modern health system as they strive to serve the adolescents to the best of their ability though in different contextual settings.

## Plain English language

Health service providers play a significant role in crafting and implementing health policies and programs that manage adolescent sexual health-related matters. Different strategies have been implemented in different country settings to improve adolescents sexual health-related outcomes. The role played by health service providers is not adequately documented to aid decision making in policy formulation and implementation. This work explores and documents the roles that modern health systems play through the specified health service provider in managing adolescent sexual health issues. The study also examines the potential for integrating modern health systems with indigenous health systems to improve adolescent sexual health matters. The study further presents ways of overcoming challenges that could derail the possible integration of the two mentioned health systems in the management of adolescent sexual health-related matters.

## Abstract (de)

**Hintergrund:**

Gesundheitsdienstleister spielen eine bedeutende Rolle bei der Ausarbeitung und Umsetzung von Gesundheitspolitiken und -programmen, die auf verschiedenen Ebenen des Gesundheitssystems mit sexuellen Gesundheitsproblemen bei Jugendlichen umgehen. Diese beeinflussen das sexuelle Verhalten und die Praktiken von Jugendlichen.

**Ziel:**

Diese Studie untersuchte die Rolle von Gesundheitsdienstleistern beim Umgang mit sexuellen Problemen von Jugendlichen und wie sich dies auf ihr sexuelles Verhalten und ihre sexuellen Praktiken auswirkt. Die Studie untersuchte die Gesundheitsdienstleister weiter, wie das indigene Gesundheitssystem in das moderne Gesundheitssystem integriert werden könnte, um ein wirksames Management von Problemen im Zusammenhang mit der sexuellen Gesundheit von Jugendlichen zu ermöglichen.

**Methoden:**

Eine qualitative Querschnittsbefragung wurde bei gezielt ausgewählten Gesundheitsdienstleistern in Gesundheitseinrichtungen in den Distrikten Mberengwa und Umguza durchgeführt. Die Datenerhebung erfolgte durch unstrukturierte Interviews, die aufgezeichnet, wörtlich transkribiert und thematisch analysiert wurden. Die Ergebnisse wurden klar definiert als über- und untergeordnete Themen präsentiert.

**Ergebnisse:**

Aus den abgefragten Daten ergaben sich insgesamt fünf übergeordnete Themen und neunzehn untergeordnete Themen. Die übergeordneten Themen waren: Überblick über sexuelle Gesundheitsprobleme bei Jugendlichen, Rolle des modernen Gesundheitssystems in Fragen der sexuellen Gesundheit von Jugendlichen, aufgetretene Herausforderungen, Faktoren des indigenen Gesundheitssystems, die in moderne Gesundheitssysteme einbezogen werden könnten, und Strategien zur Förderung der Integration des indigenen Gesundheitssystems und Modernes Gesundheitssystem. Die untergeordneten Themen vertieften die Erkenntnisse der wichtigsten Stakeholder unter den fünf übergeordneten Themen.

**Schlussfolgerungen:**

Aus den Ergebnissen kann geschlossen werden, dass Anbieter von Gesundheitsdiensten eine wesentliche Rolle bei der Gestaltung und Bereitstellung von Diensten zur sexuellen Gesundheit von Jugendlichen spielen, die Jugendliche trotz Herausforderungen nutzen, die die Nachfrage nach diesen Diensten verringert haben. Es muss daher darauf hingewiesen werden, dass es eine Gelegenheit gibt, die Zusammenarbeit zwischen dem indigenen Gesundheitssystem und dem modernen Gesundheitssystem zu fördern, während sie bestrebt sind, den Jugendlichen in unterschiedlichen Kontexten nach besten Kräften zu helfen.

## Background

Worldwide, health service providers play a significant role in crafting and implementing health policies and programs that manage health-related issues at different levels in the health system, including adolescent sexual health. These trained health service providers strive to utilise evidence-based strategies to aid efficiency and effectiveness in managing adolescent sexual health issues [1]. Despite several programs that have been implemented globally, adolescents still exhibit poor sexual health (SH) outcomes, particularly in low and middle-income countries (LMICs) [2]. Massive health service providers’ exodus hugely negatively impacts the efficiency of sexual health programs from developing to developed countries where they are better conditions of service, inadequate infrastructure to accommodate adolescents as a sensitive population, lack of training of health service providers to efficiently and effectively manage adolescent sexual health programs, shortage of drugs and medicines for adolescent sexual health-related illnesses, lack of awareness campaigns targeted at behaviour changes in adolescents and promotion of safe sexual practices in as far as adolescents are concerned [3].

Generally, health service providers play a significant role in crafting and implementing health policies and programs that manage adolescent sexual health-related matters, in turn, shaping and moulding their behaviours [4]. Due to differences in contexts, different programs are implemented in different communities to address and manage adolescent sexual health issues [5]. There are differences in how these programs are conceived, implemented and evaluated in different country settings through health service providers to ensure that these services are available [5]. Conducting such studies allows for tailor-making programs that best suit the specific country, province, or district settings to better harness the health service providers’ (HSPs) skills and explore the potential for complementary integration of different systems and ensure this translates to better adolescent sexual health (ASH) outcomes [5].

In Zimbabwe, health service providers also play a significant role in equipping adolescents with knowledge on SH, treating sexually transmitted infections, and providing different contraceptive methods [6, 7]. These services are usually available free of charge, particularly in rural areas, as the government often subsidises them [6, 7]. Despite these services’ availability, uptake of these programs remains very low, particularly in highly cultural districts where most people prefer to utilise the indigenous health system [8, 9]. It is reported that over 80% of the population in rural areas utilise Indigenous health systems (IHS) that are mannered by traditional healers and herbalists [8–10]. This then means that approximately 20% of the population utilise modern health systems (MHSs) [8]. The distribution of health facilities also impacts accessibility and subsequent utilisation of the modern health system [11, 12]. The HSPs insights relating to utilisation of health services by adolescents’ in these cultural districts considering the existence of IHS is critical in ensuring a well-coordinated and integrated health system to serve the adolescents. Therefore, it is imperative to note that HSPs play an essential role in shaping and influencing sexual behaviours and practices in adolescents that impact their SH outcomes [13, 14].

Therefore, this work sought to explore the roles played by the MHS in managing adolescent sexual health issues and appreciate the adolescent sexual health trends in the two districts. This study further challenges HSPs to identify specific indigenous health system factors incorporated into the MHS to improve adolescent sexual health outcomes. Participants were also further probed on the challenges that were likely to be encountered in integrating IHSs into MHSs and how best these could be overcome to maximise the benefits of this integration.

## Methods

### Study setting

**Fig. 1:**
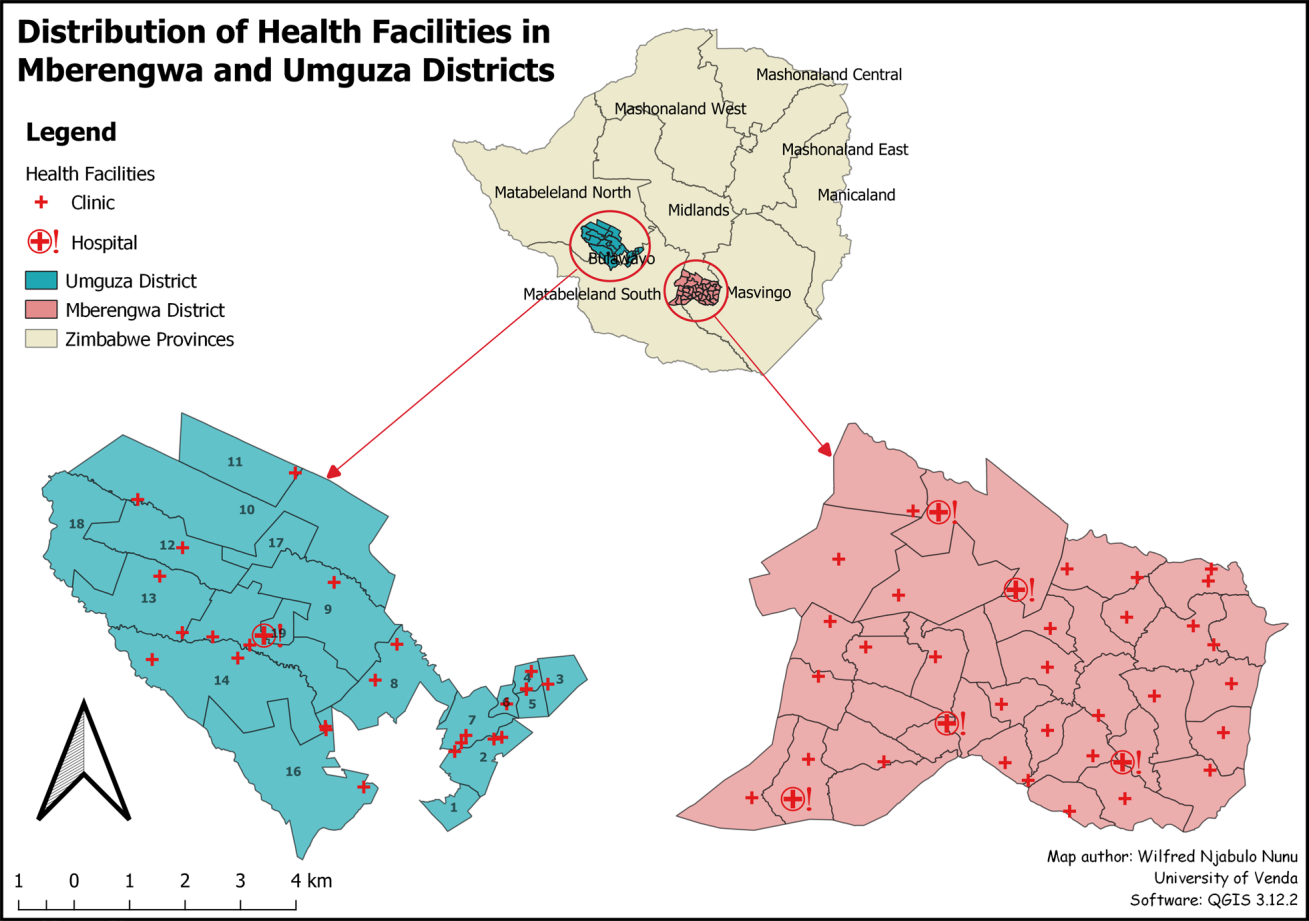
Study area map showing the distribution of health facilities in Umguza and Mberengwa districts

This study was conducted in the Umguza and Mberengwa districts of Zimbabwe. Mberengwa district has 36 health facilities, of which 31 of those are Primary Health Care (PHC) facilities, and five are referral district hospitals. 1 Umguza has a total of 26 health facilities, of which 25 are PHC facilities, and only one is a referral hospital (see Fig. 1). A PHC facility is the first point of call in a modern health system that offers essential curative and preventive care services that exclude complicated and specialised services [15–18]. The services offered in these PHC facilities are Integrated Management of Childhood Illnesses (IMCI), sexually transmitted infections (STIs)/HIV and AIDS, tuberculosis (TB), reproductive health (ante-natal care, family planning, and maternity), mental health, chronic diseases (hypertension, diabetes, and asthma), trauma and injuries and disabilities [16, 19, 20]. Any complications presented by patients in these facilities are referred to as the referral district hospitals where specialised care could be offered, failure of which the cases are further directed to central hospitals outside these two districts (Umguza and Mberengwa). The total populations in these two districts were 200,581 and 80,971 for Mberengwa and Umguza [21]. Of this population, 67,195 were aged between 5 and 14 years, 100 892 aged between 15 and 24 years in Mberengwa and 21,133 (aged 5–14 years), 47,206 (aged 15 to 24) in Umguza [8]. In simpler terms, adolescents account for over 50% of the total population in these two districts. On average, each PHC facility served a population of 6470 and 3238 in Mberengwa and Umguza, respectively. These PHC clinics are mannered by trained nurses, nurse aides, doctors (curative), Environmental Health Practitioners (EHPs) (health promotion and preventive services) [22]. Mberengwa and Umguza Districts teenage had the highest teenage pregnancy rates pegged at 17.7% and 23.6%, respectively, against the National average of 12% in 2012 [8]. These two districts still practice cultural initiations on adolescents and have a high prevalence of STIs compared to the national average [8]. Adolescent HIV and AIDS prevalence in indigenous and cultural populations are higher than the national prevalence (18% and 13.6%) [8]. These two districts are some districts with low uptake of health services and prefer to utilise the traditional health system [8, 9]. Activities engaged in as a source of income include illegal gold panning, cattle rearing, peasant farming, and migration to neighbouring countries such as South Africa and Botswana in search of employment [8, 9]. The areas classified under region five receive the lowest rainfall, making the farming business unreliable [8, 9]. Therefore the majority of the population in these areas survive on 1USD a day or less [23].

### Study approach

A qualitative cross-sectional survey was conducted on HSPs in Mberengwa and Umguza districts. A qualitative approach was chosen as it enabled the researcher to gain more insights into how the MHS operates on issues relating to ASH and understand the interaction between health service providers and the stakeholders running the IHS [24]. This qualitative research approach also enabled flexibility in probing more on issues that were not clear regarding the management of ASH-related matters by the participants, thereby allowing for a more comprehensive approach in exploring the critical problems regarding health service providers and ASH [25, 26].

### Study participants

All the 36 health facilities in Mberengwa and 26 in Umguza were targeted. From each clinic, a nurse and an EHP involved in ASH-related issues at curative, promotive, and preventive levels were targeted for recruitment into the study. A nurse, an EHP, and a doctor involved in adolescent sexual health-related issues were also targeted from each referral centre. Therefore, the sampling technique was purposive, and the sample size was determined by data saturation.

### Data collection tools

Data was collected using interviews that were recorded using a digital tape recorder. An interview guide was developed and guided the data collection process to meet the objectives. The interview guide is appended as Appendix 1 of this dissertation. Interviews were conducted either in English, Ndebele, or Shona, depending on which language the interviewee was comfortable with. The interviews took, on average, between 15 min to an hour. The interviews were conducted in private rooms in these health facilities. Prior appointments were made to minimise disturbances/interference with health facility operations. Therefore, most interviews were usually conducted in the afternoons, which were less busy than in the mornings.

### Data management and analysis

Collected data (recordings) were transcribed verbatim in Microsoft Word and, if in Ndebele or Shona, further translated to English. The English transcripts were then converted to PDF format and Imported to MAXQDA Version 14 for coding and thematic analyses. An independent coder was also engaged for expert guidance and validation of generated codes and themes. The study findings were then thematically (superordinate and subordinate themes) presented.

### Ensuring trustworthiness

This research was an output of studies towards attaining a Ph.D. in Public Health qualification for the first author guided by a published protocol [8]. The study protocol also detailed how the credibility, transferability, conformability and dependability issues were addressed in conducting the Ph.D. studies in their entirety [8]. Different committees reviewed the study protocol, and ethics approval was sought after the data collection tools were approved. Ethics clearance was given by the University of Venda’s Ethics Clearance Committee (Ethics Number: SHS/19/PH/17/2608) and the Medical Research Council of Zimbabwe (Ethics Clearance number: MRCZ/A/2611).

## Results

### Characteristics of participants

A total of 41 HSPs were recruited in this study. A total of 29 nurses/nurse aides, eight EHPs, and four doctors dealing with ASH-related issues participated in this study. More females participated as compared to males (30:11 respectively). The characteristics of the participants are presented in Table 1.

**Table 1:** Characteristics of participants

Participant type	Mberengwa Male (Female)	Umguza Male (Female)	Totals Male (Female)
Interviewed			
Nurses/nurse aides	2 (12)	1 (14)	3 (26)
Environmental Health Practitioners	3 (2)	1 (2)	4 (4)
Doctors	3 (0)	1 (0)	4 (0)
Totals	8 (14)	3 (16)	11 (30)
Grand total	41		

### Emerging themes

There was a total of five superordinate themes that emerged from interrogating the data obtained from the participants. A total of 19 subordinate themes were then obtained from the five superordinate themes, and the summary of findings is presented in Table 2.

**Table 2:** Emerging themes

Superordinate theme	Subordinate themes
Overview of ASH issues	Prevalence of STIs Prevalence of pregnancies SH-related complications Poverty
Role of MHSs in ASH issues	Education and awareness campaigns Provision of contraceptive products Treatment of STIs Performing and supervising births Performing medical male circumcision
Challenges encountered	Shortage of resources Poor health-seeking behaviour Inadequacy in the training of staff to handle adolescents Non-availability of adolescent-friendly and private facilities Promiscuous behaviours by adolescents The hostility of HSPs towards adolescents
IHS factors that could be factored into MHSs	Circumcision Humanity (Ubuntu)
Strategies to foster the integration of IHS and MHSs	Engagement between stakeholders Community cultural gatherings

#### Overview of adolescent sexual health issues

One of the superordinate themes that arose from the findings was the general overview of adolescent sexual health trends in the two districts. Under this superordinate theme, four subordinate themes emerged and are discussed in-depth in the section that follows:

##### Prevalence of STIs

The prevalence of STIs in the districts was reported to be going up. Participants felt that adolescents in these two districts generally had feeble health-seeking behaviours. Participants further cited that adolescents now engage in sexual activities early and with older people risking suffering from STIs. It was also reported that most females are more at risk from STIs than male adolescents as they are usually targeted by an older man who would have been through a lot in terms of sexual engagement.

Participants said:

*“We have observed a steep rise in the STI prevalence, particularly in female adolescents. The biggest challenge is that these adolescents have poor health-seeking behaviours as they seldom come to these facilities; only when they are now severely ill would they turn up for treatment”*. **Participant 10, Female (48 years).**

*“Very young adolescents are now presenting at the health facility suffering from STIs. The biggest challenge is that adolescents now engage in sexual activities at an early age. They date individuals who are way older than them, particularly the girls. These cross-generational sexual activities have been the major contributing factor to the high prevalence of STIs that we are observing”. Participant 11, Female (42 years).*

*“These adolescents that we treat here when they go back to the community and feel better, they throw away the medication we would have given them even though they have not completely healed, and they continue spreading it, resulting in this high prevalence of STIs”.* **Participant 1, Female (52 years).**

##### Prevalence of pregnancies

One of the subordinate themes that came up under this superordinate theme was that there are very high teenage pregnancies as several adolescents are forced to drop out of school. The HSPs felt that most pregnancies happen during the Christmas holidays when the cross-border workers, popularly known as *injiva* will be back for the festive holidays. Participants further cited that these *injiva* would be having a disposable income that there can use to lure and coerce the adolescents, particularly the females, into having sexual relations with them over those holidays. However, participants further pointed out that many illegal gold miners (popularly known as amakorokoza in the local communities) entice these adolescents into having some sexual relations with them.

One participant said:

*“We have failed as health service providers to curb adolescent pregnancies as they go berserk during the festive holidays when they are coerced by the injivas who would have saved up to come to enjoy holidays back at home. Bearing in mind that the rural setup is different from townships, adolescents have their huts which would be sometimes far from the parents' huts meaning they could sneak out easily and go and have sexual intercourse without anyone noticing”.* **Participant 28, Male (35 years).**

##### Sexual health-related complications

Participants felt that due to adolescents’ poor health-seeking behaviours and the issue of seeking treatment late, they have some complications regarding STIs left untreated for a very long time and had to refer the adolescents to Central Hospitals outside the districts for specialised care. Adolescents have also had complications during delivery as some of their pregnancies would have been discovered very late by their parents.

Participants said:

*“One of the adolescents went for circumcision, he came after he was culturally circumcised, and he was not healing. When we attended to him, he disclosed that he had had an STI for months and was discharging a lot of pus. After we enquired why he did not present to the health facilities earlier, he said he was afraid his parents would discover as they are not allowed to sleep with women before they were circumcised. The condition worsened after circumcision, and he had to be brought to the facility. We referred the case to Mpilo Central Hospital”.* **Participant 10, Female (48 years).**

*“Let me give you an experienced that shocked me. There was this adolescent girl who came with her parents complaining of stomach cramps. The parents did not know the adolescent was pregnant, only to be told when they brought her that she was in labour. The unfortunate part was that the baby was a breach, so we had to refer them to a central hospital. It was unfortunate that both the adolescent and the baby did not make it”.* **Participant 11, Female (42 years).**

##### Poverty

Participants, particularly from the Nyamandlovu area in Umguza, cited that most homesteads are mannered by adolescents as their parents and guardians migrate to South Africa and Botswana in search of opportunities to be able to fend for their families. Most adolescents are forced into sex by poverty as they try to make ends meet. However, the majority do not make much, and sometimes they cannot fully support their adolescents, leading to them engaging in sexual intercourse with older people to get basics from those people though predisposing themselves to STIs and pregnancies.

Participants said:

*“Due to financial challenges here in the rural areas, most breadwinners head to South Africa or Botswana in search of jobs to fend for their families, leaving their adolescents behind. If you realise that most are not sending any monies home because of this lockdown caused by COVID 19. How do you think these adolescents survive? Some end up dating older men and women to get favours”.* **Participant 25, Female (22 years).**

*“Adolescents struggle if they are left alone by their caregivers as they do not have a proper family structure to support them. Therefore, if they are now household heads, homestead now becomes a venue for other adolescents who live with parents to engage in sexual activities. Most parents are driven to migrate to other towns and even other countries searching for opportunities to make a living and fend for their adolescents”.* **Participant 4, Female (58 years).**

#### Role of MHS in ASH issues

Four subordinate themes emerged under the superordinate theme “Role of MHSs in ASH issues.” These are described in depth in the sections that follow.

##### Education and awareness campaigns

The key participants were involved in educating the general populace, including adolescents, on SH related issues to foster safer sexual practices in the communities. Participants further cited that they even get invited to schools, churches, and community gatherings to disseminate SH’s information to improve sexual health outcomes. Participants also noted that in partnership with different Non-Governmental Organisations (NGOs) dealing with sexual health issues, they develop flyers, training material and help ensure that the distribution processes are as effective as possible.

Participants said:

*“We are involved in education and awareness campaigns where we develop teaching material and ensure we go to schools, churches, and any community gatherings through invitation or voluntarily and disseminate information regarding adolescent sexual health-related issues.”* **Participant 14, Male (43 years).**

*“We work with different stakeholders, including NGOs, as I have said that our duty is centred on health promotion through prevention. We develop flyers, education material and deliver education and awareness sessions to promote safer sexual practices in adolescents. Therefore, we have to ensure we reach out to most adolescents and equip them with knowledge”.* **Participant 26, Male (38 years).**

##### Provision of contraceptive products

It emerged that several participants, particularly the Nurses, Nurse Aides, and Doctors, were involved in the distribution/insertion of contraceptive products. After adolescents have been educated about their services, they access these services with an informed mindset. It, therefore, emerged that several participants played a significant role in the provision of these services.

One Participant said

*“We provide contraceptives to women, including these adolescents, as well as male condoms for male adolescents as well. We usually advertise our services during the awareness campaigns so that those adolescents who decide to engage in sexual activities do so in a manner that reduces the chances of being infected as well as reduce the chances of them having unwanted pregnancies, which lead to unplanned responsibilities and resulting in high rates of school dropouts”.* **Participant 20, Female (27 years).**

##### Treatment of STIs

One subtheme that emerged under the role of these critical stakeholders was that they were involved in the treatment of STIs and providing support to those infected with HIV and AIDS through different programs and the provision of anti-retroviral therapy (ART). Participants cited that adolescents usually come to the clinics to seek treatment, although most of the time they come when the infections are at advanced stages due to some barriers. Participants also cited that they also encourage those that they treat to bring their partners to reduce reinfection.

Participants said:

*“We have been involved in the treatment of STIs over and above other roles, such as the provision of contraceptives. However, we face challenges that these adolescents come when infections are already at an advanced stage”.* **Participant 10, Female (48 years).**

*“We treat STIs and provide support services for those that are living with HIV and AIDS and also do contact tracing and encourage adolescents to bring their partners for treatment as well to minimise chances of reinfection”.* **Participant 18, Female (47 years).**

##### Performing and supervising births

It emerged that a significant proportion of adolescents come to the facilities to deliver. However, the majority are referred to Central hospitals as they are deemed at high risk. Participants cited that most of the adolescents come to the facilities already at advanced stages of labour. Participants, therefore, noted that though it is encouraged that these adolescents deliver in central health facilities with adequate infrastructure and human resources to deal with potential complications, the majority deliver in their clinics sometimes due to other factors such as transport challenges.

Participants said:

*“We supervise several adolescents giving birth though normally they are expected to be attended to in Central hospitals with the capacity to deal with complications. However, most times, these adolescents come at an advanced stage of labour with some even delivering on their way to the clinics”.* **Participant 34, Female (42 years).**

*“We are generally not expected to supervise births by adolescents as they are expected to be attended to in the higher hierarchy of our health facilities. Considering that we are in deep rural areas, there are many challenges that we face that hinder us from referring these adolescents to either district hospitals or central hospitals outside our districts, one of such being transport”.* **Participant 40, Male (36 years).**

##### Performing medical male circumcision

It also emerged that Health service providers are involved in circumcising adolescents at clinics, with some facilities partnering with NGOs to ensure that this service is available to fight against the spread of HIV and AIDS.

One participant said:

*“We are partnering with some NGOs to provide circumcision though our numbers are deficient. We are 5 months into the program, and we are failing to reach the intended targets as most of the adolescents prefer to go and get circumcised culturally”.* **Participant 3, Male (33 years).**

#### Challenges encountered

A total of six subordinate themes arose to denote challenges that are faced by the MHS and its HSPs in managing ASH related issues. These are discussed in-depth below:

##### Shortage of resources

Participants cited that different stakeholders are usually invited to participate in health awareness campaigns, particularly by the schools. However, HSPs mentioned that they are very short-staffed in clinics and very much overwhelmed and fail to respond positively to these invitations, which present an opportunity to engage with adolescents in a comfortable environment.

Participants said:

*“We usually receive invitations from schools to go and engage with adolescents or participate in awareness campaigns. We seldom go as we are very short-staffed in these clinics; for example, in this clinic, we are only two (nurses), so you can imagine that if I leave my colleague alone, how much workload would she have?” Participant 3, Male (33 years).*

*“We would have hoped to do more than we are currently doing. However, we rarely have adequate resources to cover most of the adolescents’ needs except when an NGO comes and implements a project; then, we become part of it. Usually, if that NGO pulls out after their funding is exhausted, we cannot sustain the programs”.* **Participant 30, Female (45 years).**

*“I am expected to cover two wards that amount to about 40 square kilometres to conduct awareness campaigns and do contact tracing. My motorbike broke down two years ago, and it is in a state of disrepair, as you can see there. I cannot consistently walk these 40 square kilometres; therefore, we normally do what is in our power”.* **Participant 41 Male (43 years).**

##### Poor health-seeking behaviour

One of the subordinate themes that came up under challenges was that most adolescents that belong to very cultural families have deplorable health-seeking behaviour. Participants cited that they usually delay seeking treatment until it is too late to be rushed to health facilities.

Participants said:

*“We usually conduct health education on those that would have come to the clinics with the hope that they will share the information with their peers. The biggest challenge, however, is that they usually come to the clinics when they are very ill; for example, when they are being circumcised there during their cultural initiation ceremonies in the bush, women are not allowed to go there, so we are not able to go there, the adolescent would be brought to the clinic when they see that the person is now dying”.* **Participant 41 Male (43 years).**

*“The majority of the cultures, particularly the Xhosa here, who are predominant in Ntabazinduna, rarely seek services from the clinics. They first go to the Traditional Healers and would only bring the adolescent after they would have failed of which majority of the times it would be late and a lot of damage has already been done”. Participant 10, Female (48 years).*

*“Those that have their STIs treated by the Traditional Healers some do not heal completely, and they spread that STI or they suffer severe consequences later on”.* **Participant 11, Female (42 years).**

##### Inadequacy in the training of staff to handle adolescents

Participants cited they felt a gap in terms of the knowledge and capacity to address adolescents’ issues fully. Participants felt that gap had hindered their ability to successfully lure adolescents to health facilities to seek services or even information. They felt they lack the necessary skills to deal effectively with adolescents, which has lowered the demand for their programs.

Participants said:

*“We rarely undergo training on how to deal with adolescents. They are a sensitive age group, and we need to understand how to handle them fully. We have read many developments in the field of sexual health and adolescents; it is quite challenging to keep up without being trained”.* **Participant 6, Female (42 years).**

*“In some institutions, they have dedicated Health personnel that deal with adolescent sexual health issues. However, here we are, a jack of all trades. It will have been great if some are trained to cope with the adolescents' sexual health needs”.* **Participant 10, Female (48 years).**

*“Adolescents are very delicate and need to be accommodated as they are at a stage where they are trying to discover who they are. We need to be trained on how we deal with them to foster behaviour change and adoption of safe practices”.* **Participant 41, Male (43 years).**

##### Non-availability of adolescent-friendly and private facilities

Participants cited that one of the significant challenges they encountered in the discharge of their duties was the lack of youth-friendly infrastructure in the public facilities that accorded the youths enough privacy. Participants cited that adolescents value privacy as sometimes they would seek services without their parents or guardians’ knowledge. Participants, therefore, felt that there is a need for adoption of youth-friendly facilities, such as those offered in Harare where adolescents have dedicated private rooms at facilities and use back door entries that are barricaded from the general public maximum privacy and ensure they are comfortable.

Participants said:

*“We feel our facilities are too public and do not accord adolescents the privacy that they require as they share facilities with the general populace. They are often judged if they seek contraceptive methods leading them to shun away from the facilities”. Participant 10, Female (48 years).*

*“Let us bear in mind that adolescents usually need maximum privacy if they seek sexual health services. They are typically judged harshly by the community if they are seen seeking sexual services and engaging in sexual activities. The majority would not want their guardians, parents, and elders to see them in clinics seeking sexual health services”*. **Participant 11, Female (42 years).**

*“We are not well equipped to lure adolescents into utilising our facilities leading to them engaging in unsafe sexual activities. Our facilities do not have privacy as adolescents queue together with the general populace to consult on sexual health-related issues leading to stigmatisation and judgment. In the capital city, we have special clinics for adolescents; for instance, the Mbare clinic that deals with ASH related issues. In some clinics in Epworth, they have private rooms for adolescents, and they do not use the same entrances as the general populace. We seriously need to invest very much in such strategies to ensure uptake of our programs and improve SH outcomes of adolescents”.* **Participant 30, Female (28 years).**

##### Promiscuous behaviours by adolescents

Another subordinate theme that emerged was that adolescents exhibit promiscuous behaviours and engage in sexual activities early and with multiple partners. This becomes a challenge as they are hard to control, and at the end of the day, predispose themselves to infections and unplanned and unwanted pregnancies.

Participants said:

*“These kids rush into having sexual intercourse with the majority starting from the 12 years of age, which is now common in this district. The worrying issue is that they have multiple partners and brag about it as if it is fashionable. I normally ask myself whether it is this technology that they are exposed to or not”.* **Participant 2, Female (50 years).**

*“The majority of our adolescents no longer get to 20 years without children. By that age, there would be mothers or fathers of two to children on average. They no longer go through the stages of puberty properly and fully mature. They would have been parents already; that is why we have a significant proportion of these adolescents dropping out of school”.* **Participant 41, Male (43 years).**

*“Most of these cultural initiations incite the adolescents to engage in early sexual activities as they feel they would have been given the right of passage, and they think and feel they are ready to engage in sexual activities. Most of them end up having multiple partners”.* **Participant 40, Male (36 years).**

##### Hostility of HSPs towards adolescents

It emerged that some of the health service providers have a hostile attitude towards members seeking health services, including adolescents who become intimidated. Participants cited that some members do not uphold values with isolated cases where HSPs have been reported to have physically or emotionally abused their clients. Furthermore, participants mentioned that they now give as minimum effort as possible as most are generally disgruntled with the working conditions and remuneration of their services obtaining in the country.

Participants said:

*“A long time ago, we usually thought nursing was a calling, and most individuals were not driven by the financial benefits associated with the job. I think we were confused as one needs the money and makes ends meet if they are productive. Due to the economic situation obtaining in the country, people do not work that much or do not commit themselves that much and sometimes become hostile even to the patients”.* **Participant 38, Female (32 years).**

*“You realise that the government told us to work for 2 days a week as they are not able to pay as well? However, in rural areas, we are expected to work 6 days a week, yet our counterparts work for 2 days a week, and the other 5 days, they look for employment to augment their income. Do you think we would apply ourselves that much and treat the patients fairly, yet there is that inequity?”* **Participant 10, Female (48 years).**

#### Indigenous health system factors that could be factored into modern health systems

Two subordinate themes emerged under this superordinate theme. These are discussed in-depth in the sections below.

##### Circumcision

Another theme that emerged as an essential factor that needs to be considered for adoption from the IHS is how they mobilise and get adolescents to be circumcised. Participants cited that they are facing challenges in obtaining the required numbers and meeting the targets. Participants felt that working with the IHS in the male circumcision program would ensure that they ensure the targets are met and provide the circumcision procedures done safely and effectively, whether culturally or through health facilities.

One participant said:

*“Culturally, the communities perform circumcision on the adolescents, of which that one of the strategies is we are recently trying to implement in our clinics as funded by the NGOs, and we have failed to get the numbers. When you compare with the initiation gatherings conducted in the communities, they can mobilise the numbers and ensure more adolescents are circumcised. However, we are not fully in support of how it is done. We can, therefore, team up as stakeholders and supervise these circumcisions using standardised and safe methods”*. **Participant 11, Female (42 years).**

##### Humanity (Ubuntu)

It emerged from the data that one of the factors incorporated into the MHSs is Ubuntu’s issue (treating patients with humanity). Participants cited that Ubuntu’s element in the MHSs has seriously been degraded due to prevailing contextual settings where HSPs are overwhelmed, poorly remunerated, and demotivated. Participants further highlight that Ubuntu’s element is still valued in the IHS as those that run it strive to maintain their reputation and lure as many clients as possible.

Participants said:

*“Even if their way of operation is not standardised (having exact dosages), and in some cases, us having to deal with the damage that would be done. To clarify this, sometimes, their patients are given overdoses of sexual stimulants. However, the system has its weaknesses; it is premised on Ubuntu's spirit where one is not denied treatment because they do not have adequate resources to pay. In some cases, payments are even made later in life after one has healed completely and has had the opportunity to look for money. It contrasts with our MHS where payment is usually demanded upfront for most services save for only a few services”. Participant 22, Male (29 years).*

*“Our trade (nursing) is no longer a calling. We are also struggling like everybody else because most of us have lost Ubuntu's spirit, and we are no longer going the extra mile to serve these adolescents. This is different from the IHS, where one must maintain Ubuntu's element because that brings the clients home. We still need that element in our systems to dignify our work and command the respect of the public”.* **Participant 38, Female (32 years).**

#### Strategies to foster the integration of IHS and MHS

Two subordinate themes came up on how they could integrate the IHS and MHS.

##### Engagement between stakeholders

Participants felt a need for continued engagement between the IHS and MHS through workshops as both systems serve a significant chunk of the population. Participants cited that the relations are the majority of times sour as there are competing values and interests and actors in one system view the other as a competitor that is incompetent that just takes clientele unnecessarily.

Participants said:

*“One thing that you need to understand is that I have vast experience in the two systems as I am a Nurse now though my father is a Traditional Healer. This has made me see things differently and appreciate that there is room for collaborative effort rather than working in parallel. I understand that from my experience, I managed to convince my father to seek treatment for a condition that had bedevilled him for years. Still, because of his strong beliefs in his abilities to heal himself, he did not consider modern health systems as an option. I believe we have not done much to engage our indigenous health system counterparts except when there is something wrong, and it culminates to a blame game”.* **Participant 31 Female (39 Years).**

*"We once organised a workshop with the indigenous health system personnel at some point as we realised that during their adolescent circumcision period, majority of those culturally circumcised adolescents had infections, lost a lot of blood, and some even died. After the engagement, we deployed health personnel in these initiation ceremonies to monitor the process. However, we had only to send health personnel belonging to that culture. Therefore, with continuous engagement, there is a possibility of overcoming the odds and foster this integration”.* **Participant 41, Male (43 years).**

##### Community cultural gatherings

Participants cited a need to utilise the cultural gatherings to foster and forge a good relationship between the IHS and the MHS. Participants noted that in these gatherings, most information is shared, for instance, the *Umguyo* ceremony performed by Amaxhosa to initiate male adolescents. Participants felt information sharing and sharing of values in such gatherings could also be used as a platform for collaboration.

Participants said:

*“The majority of health service providers are divorced and distance themselves from events that are going on in these communities as most do not belong to the districts. This creates segregation as there is no trust and collegiality between the key stakeholders in the communities and the HSPs. This stifles programs that we want to implement in these communities”*. **Participant 9, Male (28 years).**

*“With me, I feel integrated into the community as I grew up here and from this district. However, most of my colleagues find it difficult to fit in the societies as they do not mix and mingle with the community workers. The community also sees them as in a different class, thereby hindering collaborative efforts. A good starting point is to be involved in these community activities and adopts them as a platform for dialogue and information sharing”. Participant 12 Female (22 years).*

## Discussion

The findings of this study point out that the prevalence of STIs and pregnancies has been very high by the key stakeholders, which has led to complications during delivery and high school dropouts. It is also reported that one of the driving factors for adolescents to engage in sexual activities was fuelled by poverty. Most adolescents were left in charge in their homes as parents migrated to seek opportunities to sustain their livelihoods. These findings are well supported by different studies that have found a relationship between poverty, promiscuity, and very low ages at first sex in adolescents in resource-poor settings as individuals engage in transactional sex for livelihoods [27, 28]. Engagement in this transactional sex leads to higher chances of infection by STIs and a high prevalence of pregnancies. Adolescents cannot negotiate for safer sex as they lack the decision-making power to engage older experienced individuals [29]. Because these adolescents are still at developmental stages, the risk of complications during pregnancy and delivery is incredibly high if they get pregnant [30, 31].

It emerged from this study that health service providers play several roles in adolescent sexual health issues. These roles were centred on providing education, awareness, contraceptives, birth supervision, and conducting circumcisions. These services expect a basic package offered to adolescents as enshrined in the PHC expected minimum package [16, 19]. However, it is quite a challenge to deal with adolescents as it was reported by this study that adolescents delay seeking treatment, particularly if they are suffering from STIs. Some studies support these findings that adolescents need to feel comfortable and secure if seeking these services [32]. This could be one reason that even though circumcision services are available for them, they do not take them up and prefer to go for the culturally performed one where they feel comfortable and secure [32].

It was observed from this study that shortage of resources, poor health-seeking behaviours of adolescents, inadequately trained health service providers to handle ASH matters, hostile staff, facilities that are not friendly to adolescents, and promiscuous behaviours by adolescents as well. It has been reported by several studies that adolescents are delicate and are prone to abuse; therefore, enough resources need to be channelled towards coming up with strategies that ensure adolescents are accommodated in the health systems [33]. To elaborate; further, a significant number of adolescents have committed suicide due to failure to cope with stress, sexual abuse, and general abuse in their communities, calling for a need that HSPs are sufficiently trained to provide counselling and sufficient support services to rehabilitate these adolescents so that they are reintegrated to society after such traumatising experiences [34]. Studies have also reported poverty as the biggest driver of promiscuous behaviours in adolescents, which then predisposes them to risks of contracting and spreading STIs and unwanted pregnancies [27, 28]. Therefore, it is argued that if these conditions are addressed, this could create a progressive environment that would encourage adolescents to utilise MHSs with ease and confidence [27, 28].

Participants felt a need to adopt some of the ways in the IHS to increase the demand for services and promote collaborative efforts. These were mentioned as Ubuntu and adopting and riding on ways of mobilising adolescents for circumcision done by the IHS. Most of the time, health service providers have been accused of having a judgemental attitude towards adolescents, which is usually a deterrent for them to access services [35]. However, it is noted that the indigenous health system’s driving value seeks to leverage on Ubuntu and is not usually driven by financial gains [35]. It could be noted that the IHS survives through clientele; therefore, they are bound to treat their clients with dignity with little or no hostility reported in the modern health system [36]. Adolescents usually would engage in programs that they have trust in and where they feel value. Circumcision during their cultural initiation ceremonies, they are treated with utmost importance and value, and they feel appreciated as they move from boyhood to manhood [37]. If HSPs partner and be involved in such activities without undermining the cultural processes; they could ensure that the circumcision process is done safely [38].

This study pointed out that possible ways could be used to forge a collaborative effort between the IHS and the MHS. These were cited as promoting engagement between stakeholders in the two systems and health service providers participating in cultural gatherings. It should be noted that these proposals aid trust among different stakeholders and thus improves relations and confidence, thereby becoming a platform for the exchange of ideas and fostering teamwork and establishment of common goals that would be understood and pursued by all stakeholders with minimum opposition and resistance [38]. All stakeholders become part of one team and are bound to pull in the same direction, consult each other, and possibly attain quality outcomes as far as ASH-related issues are concerned [38].

## Strengths and limitations of the study

This paper contributed to the development of strategies that would facilitate the integration of indigenous health systems and modern health systems in the management of adolescent sexual health issues [39]. However, the study was conducted during the peak of the COVID-19 pandemic. The researchers relied in most instances on online/electronic methods of collecting data thus some areas are remote, and it was hard to get all participants to submit or participate in the study. Some areas had limited accessibility due to erratic network connectivity.

## Conclusions

It can be concluded that HSPs play an essential role in shaping and providing adolescent sexual health services utilised by adolescents despite challenges that have reduced demand for these services. Therefore, it is pertinent to note a need for collaborative efforts between the IHS and the MHS. There are complementary efforts rather than parallel efforts that could be detrimental in addressing ASH issues. There is, therefore, a need to point out that there is a window of opportunity to foster collaboration between these two systems as they strive to serve the adolescents to the best of their ability though in different contextual settings.

## Data Availability

Not applicable.

## Appendix 1: Interview guide

1. Give us the general overview of adolescent STI trends in the area that you serve?
2. Explain the role that the modern health system plays in adolescent sexual issues in your area?
3. What are the challenges acting as barriers to adolescent sexual health in your facilities concerning the modern health system?
4. What key aspects of the indigenous health system that you think could be factored into the modern health systems and the likely benefits that this integration could yield?
5. What strategies do you think should be implemented to facilitate integrating the stated aspects of indigenous health system and modern health systems in dealing with adolescent sexual health issues?
6. Do you have anything you would like to share with the researchers you feel would be useful in this research?

## Abbreviations

AIDS: Acquired immunodeficiency syndrome
ART: Anti-retroviral therapy
ASH: Adolescent sexual health
COVID-19: Coronavirus disease
EHP: Environmental Health Practitioner
HIV: Human immunodeficiency virus
HSPs: Health service providers
IHS: Indigenous health system
LMICs: Low to middle income countries
MHS: Modern health system
NGOs: Non-Governmental Organisations
PHC: Primary health care
SH: Sexual health
STIs: Sexually transmitted infections
TB: Tuberculosis

## References

[1] Ciapponi A, Lewin S, Herrera CA, Opiyo N, Pantoja T, Paulsen E, Rada G, Wiysonge CS, Bastías G, Dudley L (2017). Delivery arrangements for health systems in low-income countries: an overview of systematic reviews. Cochrane Database Syst Rev.

[2] Santhya K, Jejeebhoy SJ (2015). Sexual and reproductive health and rights of adolescent girls: evidence from low-and middle-income countries. Glob Public Health.

[3] Connell J (2010). Migration and the globalisation of health care: the health worker exodus?.

[4] Hart TL, Coon DW, Kowalkowski MA, Zhang K, Hersom JI, Goltz HH, Wittmann DA, Latini DM (2014). Changes in sexual roles and quality of life for gay men after prostate cancer: challenges for sexual health providers. J Sex Med.

[5] Giami A (2002). Sexual health: the emergence, development, and diversity of a concept. Annu Rev Sex Res.

[6] MOHCC. National Adolescent and Youth sexual and reproductive Health (ASRH) Strategy II: 2016–2020. Stepping up for good sexual and reproductive health outcomes for adolescents and youth in Zimbabwe. 2016.

[7] MOHCC. Zimbabwe National Adolescent Fertility Study, Harare. MoHCC technical report authored by Dr. Naomi N. Wekwete, Prof. Simbarashe Rusakaniko and Mr George Zimbizi (Consultants). 2016.

[8] Nunu WN, Makhado L, Mabunda JT, Lebese RT (2020). Strategies to facilitate safe sexual practices in adolescents through integrated health systems in selected districts of Zimbabwe: a mixed method study protocol. Reprod Health.

[9] Nunu WN, Makhado L, Mabunda JT, Lebese RT (2021). Indigenous health systems and adolescent sexual health in Umguza and Mberengwa Districts of Zimbabwe: community key stakeholders’ perspectives. Health Serv Insights.

[10] Nunu WN, Ndlovu V, Maviza A, Moyo M, Dube O (2019). Factors associated with home births in a selected ward in Mberengwa District, Zimbabwe. Midwifery.

[11] Mudyarabikwa O, Mbengwa A. Distribution of public sector health workers in Zimbabwe: a challenge for equity in health. In*.*: EQUINET Discussion paper 34. Harare: EQUINET; 2006.

[12] Kumaranayake L, Lake S, Mujinja P, Hongoro C, Mpembeni R (2000). How do countries regulate the health sector? Evidence from Tanzania and Zimbabwe. Health Policy Plan.

[13] Peters DH, Garg A, Bloom G, Walker DG, Brieger WR, Hafizur Rahman M (2008). Poverty and access to health care in developing countries. Ann N Y Acad Sci.

[14] Peters DH, Mirchandani GG, Hansen PM (2004). Strategies for engaging the private sector in sexual and reproductive health: how effective are they?. Health Policy Plan.

[15] Le Roux K, Le Roux IM, Mbewu N, Davis E (2015). The role of community health workers in the re-engineering of primary health care in rural Eastern Cape. S Afr Fam Pract.

[16] Mohapi MC, Basu D (2012). PHC re-engineering may relieve overburdened tertiary hospitals in South Africa. SAMJ S Afr Med J.

[17] Organization WH. Primary health care: report of the international conference on primary health care, Alma-Ata, USSR, 6–12 September 1978: World Health Organization; 1978.

[18] Chimbindi N, Bärnighausen T, Newell M (2013). An integrated approach to improving the availability and utilisation of tuberculosis healthcare in rural South Africa. S Afr Med J.

[19] Dookie S, Singh S (2012). Primary health services at district level in South Africa: a critique of the primary health care approach. BMC Fam Pract.

[20] Pascoe GC (1983). Patient satisfaction in primary health care: a literature review and analysis. Eval Program Plann.

[21] Agency ZNS (2012). Zimbabwe population census, 2012.

[22] Hongoro C, Musonza T, Macq J, Anozie A (1998). A qualitative assessment of the referral system at district level in Zimbabwe: implications on efficiency and effective delivery of health services. Cent Afr J Med.

[23] Huruva K. The socio economic benefits of community gardening as a livelihood strategy in Manquma and Cheza villages of Gokwe south (ward 14, Njelele 3) Zimbabwe. BUSE; 2015.

[24] Anderson C (2010). Presenting and evaluating qualitative research. Am J Pharm Educ.

[25] Baxter P, Jack S (2008). Qualitative case study methodology: study design and implementation for novice researchers. Qual Rep.

[26] Almalki S (2016). Integrating quantitative and qualitative data in mixed methods research-challenges and benefits. J Educ Learn.

[27] Raphael J (2015). Listening to Olivia: violence, poverty, and prostitution.

[28] Cusick L (2002). Youth prostitution: a literature review. Child Abuse Rev.

[29] Willis BM, Levy BS (2002). Child prostitution: global health burden, research needs, and interventions. Lancet.

[30] Bagley C (1999). Adolescent prostitution in Canada and the Philippines: statistical comparisons, an ethnographic account and policy options. Int Soc Work.

[31] Omar K, Hasim S, Muhammad NA, Jaffar A, Hashim SM, Siraj HH (2010). Adolescent pregnancy outcomes and risk factors in Malaysia. Int J Gynecol Obstet.

[32] Joshi BN, Chauhan S, Donde U, Tryambake V, Gaikwad N, Bhadoria V (2006). Reproductive health problems and help seeking behavior among adolescents in urban India. Indian J Pediatr.

[33] Barker G (2007). Adolescents, social support and help-seeking behaviour.

[34] Mantula F, Saloojee H (2016). Child sexual abuse in Zimbabwe. J Child Sex Abus.

[35] Epprecht M (2012). Sexual minorities, human rights and public health strategies in Africa. Afr Aff.

[36] Edwards S, Makunga N, Ngcobo S, Dhlomo M (2004). Ubuntu: A cultural method of mental health promotion. Int J Ment Health Promot.

[37] Marck J (1997). Aspects of male circumcision in subequatorial African culture history. Health Transit Rev.

[38] Themistocleous M, Mantzana V (2004). Identifying and classifying benefits of integrated healthcare systems using an actor-oriented approach. J Comput Inf Technol.

[39] Nunu WN, Makhado L. Developing strategies for integrating indigenous health and modern health systems for improved adolescent sexual health outcomes in Umguza and Mberengwa districts in Zimbabwe. 2021;14:11786329211036018. 10.1177/11786329211036018 PMC8323425 34376991

